# Safety evaluation of Phytovagex, a pessary formulation of* Nigella sativa*, on pregnant rats

**Published:** 2016

**Authors:** Reza Salarinia, Hassan Rakhshandeh, Davood Oliaee, Sima Gul Ghasemi, Ahmad Ghorbani

**Affiliations:** 1 *Department of Medical Biotechnology, Faculty of Medicine, Mashhad University of Medical Sciences, Mashhad, Iran*; 2 *Pharmacological Research Center of Medicinal Plants, School of Medicine, Mashhad University of Medical Sciences, Mashhad, Iran*; 3 *Department of Pharmacology, School of Medicine, Mashhad University of Medical Sciences, Mashhad, Iran*; 4 *Student Research Committee, Mashhad University of Medical Sciences, Mashhad, Iran*; 5 *Neurogenic Inflammation Research Centre, Department of Physiology, School of Medicine, Mashhad University of Medical Sciences, Mashhad, Iran*

**Keywords:** *Nigella sativa*, *Pregnancy*, *Rat*, *Stillbirth*

## Abstract

**Objective:**

The possible toxicity of drugs in pregnancy should be tested before their use in pregnant patients. In the present study, we aimed to evaluate the safety of phytovagex, a pessary formulation of* Nigella sativa *(*N. sativa*), which is already in clinical use for vaginal fungal infection.

**Materials and Methods:**

The pregnant rats were treated intravaginal with physiological saline (vehicle) or phytovagex pessary in the first half of their pregnancy (days 1 to 10 of gestation). Duration of pregnancy and health parameters of the newborns were recorded after parturition. Also, cytotoxicity of *N. sativa* hydroalcoholic extract was tested against ovary Cho cells.

**Results:**

The phytovagex had no significant effect on the duration of pregnancy, number of newborns, weight of neonates, and percent of stillbirth. No deformity or general behavioral abnormality was observed in neonates monitored for 30 days after birth. *N. sativa* extract had no significant effect on the viability of ovary cells at the concentrations of 12.5-200 µg/mL.

**Conclusion:**

Results of this animal study showed that phytovagex has no overall effect on the duration of pregnancy and health parameters of the newborns. Also, its active agent, *N. sativa*, does not induce any cytotoxic effect on ovary cells.

## Introduction

Natural products have always been an important source of drugs and, nowadays, they are widely used around the world. Because of their natural origin, these products have been historically considered harmless and free from side effects. However, although their adverse effects are less frequent than those of synthetic drugs, the idea that natural products are completely safe and free from unwanted effects is false (Calixto, 2000 ▶; De Smet PAGM). Plants contain numerous constituents, a number of which are toxic, such as pyrrolizidine alkaloids, digitalis, phorbol esters, etc (Calixto, 2000 ▶; Goel et al., 2007 ▶). Besides, contamination of plants with toxic metals or active chemical agents, misrecognition of plant components, and inappropriately prepared plant products would lead to the unwanted effects of these plants (Jordan et al., 2010 ▶). Hence, the potential side effects of all natural products need to be tested before their clinical applications. Special attention should be paid when a product is administrated for pregnant women, children, and geriatrics. Unfortunately, for many natural products, there is not sufficient information about the toxicity and side effects in pregnancy.

*Nigella sativa* (*N. sativa*), (black seed) as an annual herbaceous plant which belongs to family Ranunculaceae is one of the most widely used medicinal plants in Asia. *N. sativa* seed contains many bioactive compounds like alkaloids, flavonoids, fatty acids, and thymoquinone that have positive effects on curing several diseases (Shafiq et al., 2014 ▶). Pharmacological studies have demonstrated that *N. sativa* and thymoquinone exhibit a broad range of biological effects, including neuroprotective (Alinejad et al., 2013 ▶; Babazadeh et al., 2012 ▶), cardioprotective (Shafiq et al., 2014 ▶), renoprotective (Dollah et al., 2013 ▶), hepatoprotective (Mollazadeh and Hosseinzadeh, 2014 ▶), anti-cancer (Hosseini and Ghorbani, 2015 ▶.), anti-inflammatory (Hadi et al., 2015 ▶), anti-diabetic (Ghorbani, 2013a ▶; Ghorbani, 2013b ▶), and anti-microbial (Rakhshandeh et al., 2011 ▶; Forouzanfar et al., 2015 ▶) actions. Based on its anti-microbial effect, a vaginal suppository form of *N. sativa* has been recently formulated by our team and is already in clinical use for vaginal fungal infection. Although acute and subacute toxicity of aqueous, alcoholic, and chloroform extracts of *N. sativa* seeds have been investigated previously (Vahdati-Mashhadian et al., 2005 ▶), no study has yet evaluated the safety of *N. sativa* seed extracts on pregnancy when they are used topically. The aim of the present study was to determine the safety of vaginal suppository formulation of *N. sativa* on pregnant rats. In addition, the possible cytotoxicity of *N. sativa* seed hydroalcoholic extract was assessed using ovary cells.

## Materials and Methods

**Chemicals and Reagents**

High glucose Dulbecco's Modified Eagles Medium (DMEM) and fetal bovine serum were purchased from Gibco (Grand Island, NY, USA). The penicillin/streptomycin solution and 3-(4,5-Dimethyl-2-thiazolyl)-2,5-Diphenyl-2H-tetrazolium bromide (MTT) were obtained from Sigma (St Louis, MO, USA). Dimethyl sulfoxide (DMSO) was purchased from Merck (Darmstadt, Germany). Phytovagex pessary was provided by Sabz Daro Spadana (Isfahan, Iran).

**Animals**

Sexually mature, healthy male and female Wistar rats (200-250 g) were obtained from Laboratory Animals Research Center, Mashhad University of Medical Sciences, Iran. They were kept under standardized conditions (light/dark cycle of 12 h and temperature of 21–24°C) and fed with normal laboratory pellet diet and water *ad libitum*. All the animal procedures were in accordance with the ethical guidelines approved by Animal Care Use Committee, Mashhad University of Medical Sciences, Iran.

**Safety evaluation of phytovagex on pregnant rats**

Prior to mating, the female rats were isolated from the male rats for 30 days to rule out the pre-existing pregnancy. Then, each female rat was caged overnight with one fertile male animal. Confirming of mating was done by observing vaginal plug in the following morning. The presence of a vaginal plug was considered day 1 of gestation (Xia et al., 2013 ▶; Oliaee et al., 2014 ▶). The pregnant rats were randomly divided into two groups: control group (n = 10) that received 0.2 ml physiological saline intravaginally as vehicle and treatment group (n = 10) that received 140 mg/kg (about 1/100 piece) of phytovagex pessary. Phytovagex was given intravaginally from day 1 to day 10 of gestation (early period of organogenesis). Because each phytovagex pessary has 2.5 g weight (clinical dose is approximately 2.5 g per 70 kg body weight or about 35 mg/kg), dose of 140 mg/kg used in this study was equivalent to approximately 4 pessaries per patients. In both groups, the animals were housed individually until parturition. Duration of pregnancy, number of neonates, weight of neonates, and percent of stillbirth were recorded. 

**Preparing** ***N. sativa*** **extract**

The seeds of *N. sativa* were cleaned and grounded to the fine powder by a blender. Then, the macerated extract was prepared by the suspension of 100 g of the powder in 300 ml of 70% ethanol. The suspension was maintained at 40°C for 48 h (Shafiee-Nick et al., 2012 ▶). Then, the hydroalcoholic extract was filtered through Whatman No.1 filter paper, centrifuged for 3 min at 1500 rpm, and dried in the oven at 40ºC. The resulting extract (yield 15 g, 15% w/w) was kept at -20 ºC until use. For cytotoxicity assessment, the working solution was prepared freshly by dissolving 100 mg extract in 1 ml DMSO and dilution with DMEM (the final concentration of DMSO was ≤ 0.8%).

**Cytotoxicity assessment of** ***N. sativa*** **extract**

Chinese hamster ovary (Cho) cells were seeded in 96-well plate and cultured for 24 h in DMEM containing 10% FBS, 100 units/ml penicillin, and 100 µg/ml streptomycin. Then, the culture medium was changed to the fresh one containing vehicle (0.8% DMSO) or 12.5-400 µg/ml of *N. sativa* extract. The cells were further incubated for 24 h in an atmosphere of 5% CO_2_-95% air at  37˚C. Then, the cell viability was determined using MTT assay.

**Cell viability assay**

At the end of the incubation with *N. sativa* extract, 10 µl of MTT solution (5 mg/ml) was added to 100 µl cell culture medium of the each well. Then, the 96-well plate was incubated for 2 h at 37˚C and 5% CO_2_. Then, the cell culture medium was removed and the resulting formazan of each well was dissolved in 100 μl DMSO. The optical density of formazan was read at 545 nm (against 620 nm as background) using a microplate reader and viability was calculated as the percentage of untreated cells (Ghorbani et al., 2014 ▶; Ghorbani et al., 2015 ▶).

**Statistical analysis**

Data of cell viability assay were analyzed using one-way analysis of variance (ANOVA) followed by Tukey's post-hoc test for multiple comparisons. For the analysis of data collected from the animal study *t*-test was used. The values were reported as mean ± SEM and the p-value less than 0.05 was considered asstatistically significant. 

## Results

**Effect of** **phytovagex** **on reproduction**

**Figure 1:**
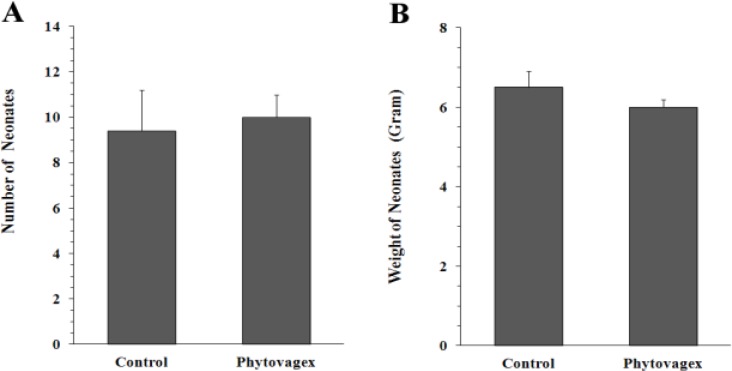
Effect of phytovagex, a pessary formulation of *N. sativa*, on the number (A) and weight (B) of newborns in pregnant rats. The pregnant animals on days 1-10 of gestation were treated with phytovagex. Values are mean ± SEM (*n* = 8

As shown in Table 1, phytovagex had no significant effect on the duration of pregnancy. Also, this drug had no significant effect on the number of rats who showed stillbirth (12.5% in both control and treatment groups). Figure 1A demonstrates that the average number of neonates in the control group (9.4 ± 1.8) was close to that of treated animals (10 ± 1). Likewise, in phytovagex treated group, weight of neonates on parturition day (6.5 ± 0.4 g) did not show any significant difference when compared with control group (6 ± 0.2 g) (Figure 1B). 

**Table 1:** Effect of phytovagex, a pessary formulation of *N. sativa*, on the duration of pregnancy and stillbirth in rats. The pregnant animals on days 1-10 of gestation were treated with phytovagex. Values are mean ± SEM

	**Control**	**Phytovagex**
**Number of pregnant rats**	8	8
**Duration of pregnancy (Day)**	23.2 ± 0.2	22.7 ± 0.2
**Number of pregnant rat** **s which showed stillbirth (%)**	1 (12.5)	1 (12.5)

Neonates in control and treatment groups were followed for 30 days after birth. No deformity or abnormality in movement and general behavior (e.g. excitement, sleepy, abnormal posture, and respiratory) was observed in both groups.

**Effect of** ***N. sativa*** **extract on cell viability**

**Figure 2:**
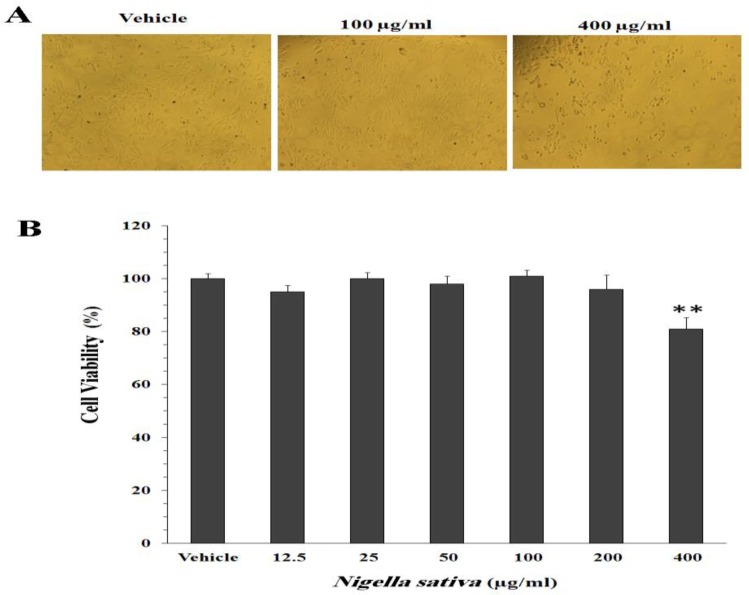
Effect of *N. sativa* hydroalcoholic extract on the proliferation of ovary Cho cells. The cells were cultured for 24 h in the medium containing vehicle (0.8% DMSO) or 12.5-400 µg/ml of the extract. A: Phase-contrast light microscopic picture of Cho cells after 24 h of treatment with vehicle, 100 µg/ml, and 400 µg/ml of the extract (Magnification: 100×). B: Percent of cell viability as determined by MTT assay. Values are mean ± SEM (*n* = 6

Figure 2A shows the effect of *N. sativa* hydroalcoholic extract on the proliferation of ovary cells. In the presence of 12.5, 25, 50, 100, and 200 µg/ml of the extract, percent of viable cells was 95 ± 2.6, 100 ± 2.3, 98 ± 3, 101 ± 2.2, and 96 ± 5.4, respectively, as compared with the untreated cells (100 ± 2%). The extract induced toxicity against Cho cells only at the concentration of 400 µg/ml (81 ± 4.4%, p<0.01). The phase-contrast microscopic pictures of the cells treated with the extract are shown in Figure 2B. 

## Discussion

It is estimated that drugs are responsible for approximately 1% of congenital anomalies of known etiology (De Santis et al., 2004 ▶). Therefore, any drug should be used during pregnancy only if adequate information is provided regarding its safety. The final conclusion on the safety of drugs on pregnancy can only become available after performing extensive epidemiological studies. Yet, basic information about the possible reproductive and developmental toxicity of a new drug can be obtained from the outcome of laboratory animal experiments (Peters et al., 2015 ▶). In the present study, we aimed to evaluate the safety of phytovagex for pregnant animals. This drug is a pessary formulation of* N. sativa* and is already in clinical use for vaginal fungal infection. In this study, phytovagex was placed in the vagina of animals from conception to day 10 of gestation, which was comparable with the time of implantation and early organogenesis period (Korgun et al., 2003 ▶; Sun et al., 2002 ▶; Oliaee et al., 2014 ▶). Organogenesis is a critical phase for the induction of congenital anomalies, functional impairments, and fetal death by pharmacological agents (Peters et al., 2015 ▶).

In the present study, duration of pregnancy was about 22-23 days which was comparable with that of our previous work (Oliaee et al., 2014 ▶) and other published reports (Wan Ezumi et al., 2007 ▶). Data of this study showed that phytovagex had no effect on the normal length of pregnancy and would not be expected to cause prematurity and dysmaturity. Also, phytovagex had no overall effect on the number of newborns, weight of newborns, and percent of stillbirth, indicating that the drug was nontoxic to the fetuses. In addition, no deformity or abnormality in movement was observed in the neonates of phytovagex treated rats, showing that there were no congenital skeletal malformations attributed to this drug.

Although this work was the first one in investigating the safety of phytovagex on pregnant rats, there are limited reports on the possible reproductive toxicity of *N. sativa* (Yadav and Agarwal, 2011 ▶; Keshri et al., 1995 ▶). Yadav and Agarwal (2011) ▶ reported that 40 days of treatment of non-pregnant rats with aqueous extract of *N. sativa* seeds dysregulated estrous cycle and led to decrease in the weight of reproductive organs; ovaries, uterus, and vagina. Keshri et al. (1995) ▶ reported that pregnancy is prevented in rats treated orally with 2 g/kg of *N. sativa* hexanic extract on days 1-10 post-coitum. Difference in extract type, limited absorption of *N. sativa* from phytovagex pessary into circulation, and use of a high dose of extract by Keshri et al. may explain different results which were obtained by these authors from those of our study. Thymoquinone, p-cymene, carvacrol, 4-terpineol, and sesquiterpene are of the main chemical composition of *N. sativa* (Nickavar et al., 2003 ▶). It has been reported that single intraperitoneal administration of thymoquinone at the dose of 35 mg/kg (but not 15 mg/kg) has a disruptive effect on embryonic development during the second trimester of rat pregnancy (AbuKhader et al., 2013 ▶). On the other hand, Al-Enazi (2007) ▶ showed that thymoquinone inhibits rate of embryo malformations during pregnancy in diabetic mice by reducing the free oxygen radicals. Therefore, further works are needed to draw final conclusion about the maternal and embryonic toxicities of thymoquinone.

In order to evaluate the potential cytotoxic action of *N. sativa* on reproductive organs, viability of ovary Cho cells was evaluated through MTT assay. The hydroalcoholic extract of *N. sativa*, even at high concentration of 200 µg/ml did not decrease the viability of ovary cells. Therefore, it seems that use of phytovagex was accompanied by no cytotoxicity. However, Yadav and Agarwal (2011) ▶ reported that the ovaries of *N. sativa* treated animals showed some cellular changes such as hypertrophy of the theca follicili, atrophic changes in the developing oocyte, and destruction of the basement membrane separating the zona granulosa from the theca folliculi. 

In conclusion, the present findings indicated that use of phytovagex in the first half of pregnancy has no overall effect on the duration of pregnancy, delivery, external malformations, and early pup growth. Also, its active agent, *N. sativa*, does not induce any cytotoxic effects on ovary cells.
